# Impact of Moderate Wine Consumption on Type 2 Diabetes

**DOI:** 10.3390/nu18122006

**Published:** 2026-06-20

**Authors:** Attilio Giacosa, Josep Masip, Ursula Fradera, Ramon Estruch, Mariangela Rondanelli

**Affiliations:** 1 Department of Gastroenterology, Italian Diagnostic Center (CDI), 20147 Milan, Italy; 2 Research Direction, Consorci Sanitari Integral, University of Barcelona, 08906 Barcelona, Spain; jmasip@ateneu.ub.edu; 3 Wine Information Council, 1040 Brussels, Belgium; ufradera@googlemail.com; 4 Department of Internal Medicine, August Pi Sunyer Biomedical Research Institute (IDIBAPS), Hospital Clinic, University of Barcelona, 08036 Barcelona, Spain; restruch@ub.edu; 5 Institut de Recerca en Nutrició i Seguretat Alimentaria (INSA-UB), University of Barcelona, 08921 Barcelona, Spain; 6 Centro de Investigación Biomédica en Red Fisiopatología de la Obesidad y la Nutrición (CIBEROBN), Institute of Health Carlos III, 28029 Madrid, Spain; 7 Department of Public Health, Experimental and Forensic Medicine, University of Pavia, 27100 Pavia, Italy; mariangela.rondanelli@unipv.it

**Keywords:** wine, alcohol, type 2 diabetes, moderate alcohol consumption, cardiovascular risk, mediterranean diet, polyphenols, cardiometabolic health, narrative review

## Abstract

Type 2 diabetes (T2D) is a prevalent disease worldwide that increases the risk of cardiovascular (CV) complications, disability and mortality. While excessive alcohol consumption is harmful, the effects of moderate wine consumption remain debated. This review evaluates whether moderate wine intake affects the risk of developing T2D and its impact on subjects with T2D. Twenty-eight studies were analysed. Evidence suggests an association between moderate wine consumption and the risk of developing T2D, with a J-shaped relationship, and reduced risk observed at low levels. This effect appears more pronounced with red wine, likely related to its higher polyphenol content, and when consumed with meals. On the other side, in patients with T2D, moderate wine consumption has been associated with a reduced risk of CV complications, nephropathy and mortality. It has also been linked to improved lipid profiles and reduced inflammatory markers, without adversely affecting body weight or glycaemic control in well-managed patients. These effects may be enhanced within a Mediterranean dietary pattern, suggesting synergistic actions. However, alcohol intake may increase the risk of hypoglycemia, particularly in patients receiving glucose-lowering therapies. It should be avoided by vulnerable individuals, and those with comorbidities such as MASLD and other significant liver diseases, peripheral neuropathy or other severe conditions. In conclusion, moderate wine consumption may be associated with a reduction in the risk of developing T2D and with several CV benefits in patients with T2D. Vulnerable patients should abstain and individuals who currently do not drink alcohol should not start drinking. If wine is consumed, intake should always remain moderate (as low as possible), within healthy meals and only after individual clinical assessment.

## 1. Introduction

Diabetes is a metabolic disease primarily characterized by elevated blood glucose levels. Its global prevalence continues to rise, representing a major public health challenge. This disease is associated with increased morbidity and mortality in both men and women, largely due to a higher risk of ischemic heart disease and stroke [1]. According to the Global Burden of Disease Study, approximately 529 million individuals had diabetes in 2021, with projections estimating a marked increase in the coming years, with 1.17 billion people with diabetes expected in 2050 [2]. The economic burden of diabetes is also substantial. It has been estimated that in 2010, healthcare expenditures related to diabetes accounted for approximately 12% of global healthcare spending, a proportion expected to increase considerably in the next 20 years. Type 2 diabetes (T2D) accounts for more than 90% of all cases of diabetes and represents a growing concern in both medical and social contexts, driven by increased lifespan, urbanization and changes in lifestyle, including dietary habits, physical inactivity and behavioral changes [3,4].

Given the critical impact of diabetes on health, considerable attention has been devoted to preventive strategies, particularly healthy dietary habits, physical activity, and body weight management [5]. While the detrimental effects of excessive alcohol consumption, including wine, are well established, the potential health effects of moderate wine consumption remain debated [6,7,8,9,10,11]. This is particularly relevant when wine is a traditional component in some Mediterranean dietary patterns [6].

This narrative review aims to examine the impact of moderate wine consumption on T2D, focusing on the role of wine within the broader Mediterranean dietary pattern. Specifically, this review addresses whether moderate wine consumption can modulate the risk of developing T2D and explores how and under what conditions patients with T2D may safely consume wine.

## 2. Materials and Methods

### 2.1. Literature Search Strategy

A comprehensive literature search was conducted across PubMed/MEDLINE, Web of Science, Cochrane Library, Embase, Scopus, and Google Scholar to identify studies examining the relationship between wine consumption and T2D. The search strategy utilized Boolean operators using the following MeSH terms and keywords: (“alcohol” OR “wine”) AND (“Type 2 diabetes” OR “T2D”).

**Figure 1:**
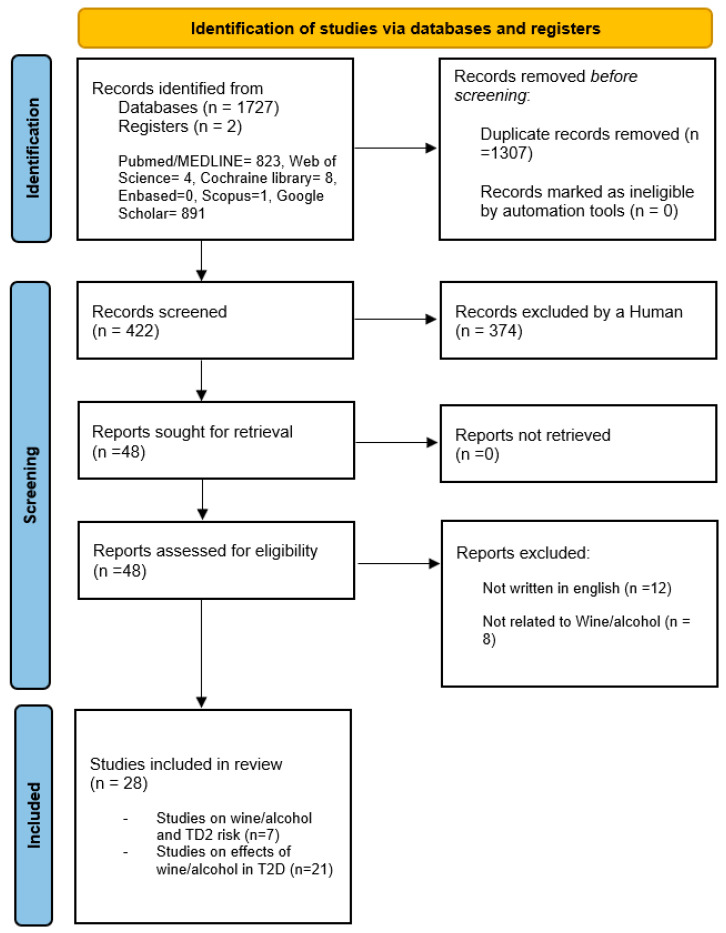
PRISMA 2020 flow chart of the literature search.

Study selection was performed in accordance with the SANRA (Scale for the Assessment of Narrative Review Articles) guidelines [12,13,14,15]. The initial results were screened to remove duplicates, followed by an evaluation of titles and abstracts for relevance. The overall selection process is summarized in the PRISMA 2020 flow diagram (Figure 1).

### 2.2. Inclusion and Exclusion Criteria

To be eligible for inclusion, studies had to meet the following criteria:

- Study Type: Peer-reviewed original research articles, systematic reviews, meta-analyses, and authoritative editorials.
- Language: Full-text articles published in English.
- Timeframe: Primary focus was placed on studies published between 1 January 2000, and 31 December 2025. However, seminal papers published prior to 2000 were included if they provided foundational evidence or critical historical context.
- Focus on Consumption Levels: A key inclusion criterion was a clear definition of “moderate consumption” within the study (typically 1–2 glasses per day or 10–30 g of ethanol). Studies addressing alcohol abuse or binge drinking were analyzed separately to assess contrasting associations with T2D risk.
- Exclusion criteria: Articles were excluded if they were not strictly relevant to the relationship between moderate wine consumption and T2D, or if the full text was unavailable.

### 2.3. Analysis of the Reports: Data Extraction and Synthesis

The narrative synthesis was structured to answer two core clinical questions:

- Does moderate wine consumption modulate the risk of developing T2D?
- Can patients with established T2D safely consume wine in moderation?

The extracted evidence was critically analysed to distinguish the effects of moderate wine intake from those of both abstention and excessive alcohol consumption. Relevant data from the selected studies (including author, year of publication, study design, and key findings) were systematically extracted and organized into summary tables to support a qualitative narrative synthesis of the evidence.

## 3. Results

### 3.1. Association Between Alcohol/Wine Consumption and the Risk of Developing Type 2 Diabetes

The association between alcohol consumption, particularly wine, and the risk of developing T2D has been extensively investigated in epidemiological research over recent decades.

We analyzed a total of seven studies: six population-based prospective studies and one meta-analysis. A detailed description of the studies included is provided in Table 1.

In 1988, Stampfer et al. published data from the Nurses’ Health Study, which included 85,051 US women [16]. Among 526 incident cases of T2D, moderate alcohol consumption was associated with a reduced risk of diabetes. However, as moderate drinkers also exhibited lower body weight, the authors concluded that it was not possible to determine whether moderate alcohol consumption independent of weight loss influenced T2D risk. More recently, a large study conducted by the same research group at the Harvard School of Public Health in Boston analyzed 200,969 subjects from three US cohorts (Nurses’ Health Study, Nurses’ Health Study II and Health Professionals follow-up Study) following them for over three decades [17]. This new data identified 20,551 cases of T2D and demonstrated that moderate alcohol consumption was associated with a significant reduction in the risk of T2D in both men and women. Notably, the favorable association was more strongly related to drinking frequency than to total quantity. In fact, risk reduction was observed from as little as 1–2 drinks per week in women and 3–4 in men, becoming more pronounced with higher frequency (>5 drinks per week), regardless of daily alcohol intake. However, the type of cohorts analyzed, composed exclusively of nurses and health professionals, may limit generalizability. In the Australian cohort studied by Hodge et al. (2006), total alcohol intake was associated with reduced risk of T2D only in women after adjustment for BMI and waist-to-hip ratio [18]. However, moderate wine consumption was the only beverage for which an inverse association was observed for both sexes.

The large European EPIC study (2012) [19] included 455,680 participants aged 35 to 70 years, across eight countries, with a mean follow-up of 9.9 years and over 12,000 incident cases of T2D. This cohort included subjects from 26 centers in Denmark, France, Germany, Italy, the Netherlands, Spain, Sweden, and the U.K. The authors found a reduced risk of T2D among women consuming moderate quantities of alcohol, but not among men. The authors reported that the differences observed between men and women could be explained by the different amount and distribution of fat mass in both sexes and by the different types of alcoholic beverages consumed. In fact, the favorable association was more evident in overweight subjects and was particularly linked to wine or fortified wine.

**Table 1:** Association between wine drinking and risk of T2D.

Study Design	Results	Effects of Wine	Reference
**Meta-analyses**			
Meta-analysis of 13 prospective studies with a total of 397,296 participants and 20,641 cases of T2D	U-shaped relationships of wine, beer and spirits with the risk of T2D. A moderate dose of wine such as 20–30 g/day led to the peak reduction of 20%. All levels of wine consumption (low: <10 g/d, moderate: 10–20 g/d and high: >20 g/d) were associated with a decreased risk of T2D	Wine appeared to be most clearly associated with a reduced risk of T2D	Huang et al., 2017 [8]
**Cohort Studies**			
Prospective study of 85,051 women (USA); 526 T2D	Compared with nondrinkers, women consuming 5–14.9 g of alcohol per day (about 4–10 drinks per week) had an age-adjusted relative risk of diabetes of 0.4 (95% CI: 0.3, 0.6); for 15 g or more per day, the relative risk was 0.3 (95% CI: 0.2, 0.4). Strong inverse association between alcohol drinking and body weight	Wine equally associated with weight loss and reduced T2D risk	Stampfer et al., 1988 [16]
Prospective study of 36,527 adults (Australia); 362 T2D	Moderate wine consumption was associated with a significant reduction in the risk of T2D for both sexes	Wine associated with reduced risk of T2D	Hodge et al., 2005 [18]
EPIC prospective Study of 455,680 adults (Europe); over 12,000 T2D	Amongst women, moderate alcohol consumption was associated with a lower incidence of diabetes with a HR of 0.82 (95% CI: 0.72, 0.92) for 6.1–12.0 g/day	Wine and fortified wine consumption appeared to be most clearly associated with a reduced risk of T2D	Beulens et al., 2012 [19]
Nord-Trøndelag Health Survey (HUNT) study of 90,296 adults; 1841 T2D	Moderate alcohol consumption was associated with a reduced risk of T2D in men, but not in women (hazard ratio for men 10–15 g/day 0.48, 95% CI: 0.28, 0.77; hazard ratio for women ≥ 10 g/day: 0.81, 95% CI: 0.33, 1.96). The reduced risk was primarily linked to consumption of wine [HR: 0.93 (95% CI: 0.87, 0.99) (per g/day)]	Wine most strongly associated with a reduced risk of T2D	Rasouli et al., 2012 [20]
312,388 current drinkers from the UK Biobank; 8598 incident cases of T2D	Consuming alcohol with meals was significantly associated with a 12% lower risk of T2D (HR: 0.88; 95% CI: 0.83, 0.93) than was consuming alcohol outside of meals	The beneficial associations between alcohol drinking with meals and lower risk of T2D were mainly driven by wine consumption	Ma et al., 2022 [7]
Prospective study of 200,969 US man and woman; 20,551 T2D	Alcohol consumption was associated with a lower risk of T2D, with either nondrinkers or 0.1–4.9 g/day as the reference. The association was robust to extended latency periods and alternative modeling of exposure. Higher drinking frequency was associated with a lower T2D risk. Compared with drinking 1–2 days per week, the HRs for drinking 5–6 days were 0.73 (95% CI: 0.65, 0.83), 0.73 (95% CI: 0.62, 0.86), and 0.76 (95% CI: 0.67, 0.86) in the NHS, NHSII, and HPFS cohorts, respectively	Association between higher drinking frequency and lower risk of T2D. No separate analysis was performed for different types of alcoholic beverages	Li et al., 2025 [17]

In 2013, Rasouli et al. analyzed 1841 patients with T2D identified among the 90,296 subjects of the Nord-Trøndelag Health Survey [20] and reported that wine consumption, but not beer or spirits, was associated with a reduced risk of T2D. The study also considered drinking patterns, including quantity and frequency of alcohol consumption, type of alcoholic beverage predominantly consumed, binge drinking, and alcohol use disorders.

The meta-analysis by Huang et al. (2017) [8] evaluated the correlation between consumption of different types of alcoholic beverages and risk of T2D. They included 20,641 patients with T2D identified from 13 prospective studies for a total of 397,296 participants. The study observed a J-shaped relationship between beer, wine, and spirits consumption and T2D risk. Wine showed a stronger favorable association compared to beer and spirits. Low (<10 g/day), moderate (10–20 g/day) or high (>20 g/day) wine consumption was associated with a reduced risk of developing T2D. Moderate wine consumption appeared to be the dose most favorably associated with the reduction in the risk of T2D (20% reduction). The authors concluded that, while confirmation through randomized intervention studies is needed, there is epidemiological evidence of an advantage of wine over beer and spirits in preventing the risk of T2D. The authors hypothesized that the polyphenolic content of wine may play an important role in the favorable association between wine on T2D risk.

In 2022, Ma et al. [7] examined 312,388 regular consumers of alcoholic beverages within the UK Biobank who were free of T2D at baseline and identified 8598 incident cases of T2D. The study found that consumption of alcoholic beverages with meals significantly reduced the risk of T2D (<12%) compared to consumption outside of meals, with this effect largely driven by wine consumption. Additionally, lower levels of C-reactive protein were observed among individuals who habitually consumed wine during meals, suggesting a potential anti-inflammatory mechanism.

Overall, a comparative analysis of all the studies currently available on the association between alcohol and wine consumption and the risk of developing T2D remains challenging due to heterogeneity in studies design, populations, definition of moderate alcohol consumption, type of alcoholic beverage consumed, different gender response, drinking and dietary patterns, and lifestyle factors. Nevertheless, two consistent conclusions emerge. First, low to moderate alcohol consumption in general does not increase the risk of developing T2D. Second, moderate wine consumption seems to be associated with a reduced risk of T2D, particularly when consumed with meals.

### 3.2. Effects of Moderate Wine Consumption in Patients with T2D

The effects of alcohol consumption, particularly wine, on patients with T2D remain a subject of debate. Numerous studies over the last three decades have investigated this relationship across different populations, regardless of the type of alcoholic beverage predominantly consumed. We analyzed a total of twenty-one studies: seven population-based prospective studies, one retrospective cohort study, one cross-sectional study, four meta-analyses, four randomized controlled trials (RCT), one randomized controlled crossover study and three case–control studies. A detailed description of the included studies is provided in Table 2.

A particularly interesting study was conducted by Solomon et al. (2000) in the US using data from the Nurses’ Health cohort [21]. Their analysis of women with diabetes showed that those who consumed alcoholic beverages in moderation (0.1–15 g/day) had a lower risk of developing fatal or nonfatal coronary heart disease (CHD) compared to abstainers.

Another study conducted in Switzerland in 2003, involving 287 patients with T2D as part of the WHO Multinational Study of Vascular Disease in Diabetes, evaluated the risk of CHD mortality and all-cause mortality [22]. In this cohort, moderate alcohol consumption (16–30 g/day) was associated with a significant reduction in both CHD mortality and all-cause mortality. However, alcohol consumption above 30 g/day was associated with a loss of this protective effect and a trend toward increased all-cause mortality.

In China, a 10-year follow-up, from 2011 to 2021, of 3521 patients with T2D or prediabetes revealed 227 deaths, 296 incident strokes, and 445 new cases of CHD [23]. In this population, occasional alcohol consumption (less than once a week) was associated with a reduced risk of all-cause mortality. In contrast, consumption of >15 g/day for women and >30 g/day for men was associated with an increased risk of incident stroke.

A case–control study by Pitsavos et al. (2005), involving 412 Greek patients with T2D, confirmed the relationship between alcohol consumption and ischemic heart disease [24]. The findings supported a J-shape association. Indeed, while low–moderate alcohol (<12 g/day) consumption was associated with a reduced risk of acute coronary events, high consumption was linked to an increased dyslipidemia and hypertension and a greater risk of acute coronary syndrome.

In the SMART study, which followed 5447 patients with clinically manifest vascular disease or diabetes, Beulens et al. observed that moderate consumption of alcoholic beverages (1–2 drinks per day) was associated with a significantly lower risk of both vascular and all-cause mortality. Furthermore, this moderate intake was linked to a reduced risk of non-fatal CVD events, including coronary heart disease (HR 0.39), stroke (HR 0.67), and amputations (HR 0.29). Notably, similar favorable associations were observed when red wine consumption was analyzed separately [25].

A recent nationwide Korean study conducted by Lee et al. (2025) involving 2,642,359 patients with T2D identified a J-shaped relationship between alcohol consumption and both all-cause mortality and cancer mortality [26]. Importantly, this study revealed significant heterogeneity across subgroups. Among older individuals and patients with kidney disease, higher alcohol consumption was inversely associated with all-cause mortality compared to non-drinkers. In the young and middle-aged groups, alcohol consumption, including heavy drinking, was associated with a reduced risk of cancer mortality. In contrast, among women, a linear relationship was observed between alcohol consumption and cancer mortality risk. Finally, a cross-sectional analysis based on data from the NHANES survey reported an inverse association between moderate drinking and the risk of diabetic kidney disease (DKD), whereas heavy drinkers showed an increased risk [27]. These findings suggest that moderate alcohol consumption may not adversely affect kidney function in patients with T2D and may even show association with favorable effects.

Taken together, these studies suggest a potentially favorable association between moderate alcohol consumption and patients with T2D. However, it is essential to account for patient heterogeneity, particularly with respect to age, sex, and the presence of comorbidities. Notably, many of these studies did not differentiate between types of alcoholic beverages (wine, beer, and spirits). This distinction, especially the specific effects of wine, has been addressed by the subsequent investigators. Blomster et al. (2014) analyzed 3314 patients with T2D over a 5-year follow-up and found that, compared to abstainers, moderate consumers of alcoholic beverages experienced fewer major CV events, fewer microvascular complications, and lower all-cause mortality [28]. These benefits appeared more pronounced among individuals who predominantly consumed wine.

The CASCADE (CArdiovaSCulAr Diabetes & Ethanol) trial represents the first randomized controlled trial (RCT) specifically designed to evaluate the effects of red and white wine in patients with T2D. In the initial study by Gepner et al. (2015), 224 patients were randomized to consume 150 mL of mineral water, white wine and red wine with dinner over a 2-year period, all within a Mediterranean dietary framework [29]. Compared with water or white wine, red wine consumption resulted in significant increases in high-density lipoprotein cholesterol (HDL-C) (+0.05 mmol/L), apolipoprotein A1 (+0.03 g/L), and a reduction in the total cholesterol/HDL-C ratio. Improvements in glycemic control were observed only in individuals carrying the alcohol dehydrogenase allele ADH1B*1 (slow ethanol metabolizers). Notably, these benefits on glycemic control were evident for both white and red wine, in contrast to fast ethanol metabolizers, homozygous for ADH1B*2. These findings suggest that the favorable association between wine consumption and glucose metabolism may be primarily attributable to its ethanol component rather than to beverage-specific constituents. The authors concluded that moderate wine consumption (150 mL), particularly red wine, appears safe for well-controlled patients with T2D and may confer cardiometabolic benefits, potentially mediated by both ethanol and polyphenol compounds.

The second CASCADE analysis by Golan et al. (2017) evaluated the effect of moderate consumption of red and white wine (150 mL) versus water on visceral adiposity in 48 patients with T2D [30]. A 2-year consumption of wine with dinner within a Mediterranean dietary pattern did not result in weight gain or increased abdominal adiposity [30].

Similarly, Marfella et al. (2006) conducted an RCT in 115 patients with T2D with a history of non-fatal myocardial infarction and reported that moderate red wine consumption (118 mL/day, ~11 g alcohol) over one year reduced oxidative stress, decreased pro-inflammatory cytokines, and improved cardiac function [31]. Supporting these findings, the meta-analysis by Naame et al. (2019) concluded that moderate wine intake in patients with T2D was associated with reduced blood pressure and increased HDL cholesterol, without significant changes in BMI, total cholesterol, and LDL cholesterol [32]. The randomized crossover study by Mori et al. (2016), involving 24 patients with well-controlled T2D found that moderate red wine consumption (24 g of alcohol/day for women and 31 g of alcohol/day for men) for four weeks did not significantly affect glycemic control or major CV risk markers (24 h ambulatory blood pressure and heart rate, HDL-C, fibrinogen, C-reactive protein, and homocysteine) compared with water or dealcoholized red wine [33]. During red wine consumption, an increase in blood pressure upon awakening and a decrease during sleep was observed compared to water, with a similar trend for dealcoholized red wine. Furthermore, neither red wine nor dealcoholized wine significantly affected glycemic control or other CV risk factors. The authors concluded that, in patients with well-controlled T2D, moderate red wine consumption does not significantly modify CV risk factors. However, further studies, particularly those with longer intervention periods, are needed to better elucidate the effects of wine on CV risk. The meta-analysis by Ye et al. highlighted that moderate wine consumption in patients with T2D led to a reduction in diastolic blood pressure and total cholesterol, while no changes in systolic blood pressure, glucose parameters and other CV risk indices were observed [34].

An important clinical concern is the risk of hypoglycemia associated with alcohol intake in patients with T2D. Evidence indicates that alcohol abuse is a significant risk factor for severe hypoglycemia in patients with T2D treated with insulin [35]. Patients with alcohol use disorders (AUDs) also showed an increased risk of CV events and neuropathy [36]. Conversely, a meta-analysis by Hirst et al. [37] found no significant impact of light-to-moderate alcohol consumption on glucose metabolism or severe hypoglycemic events in randomized trials, regardless of whether alcohol was consumed with or outside meals.

Certain subpopulations may be particularly vulnerable to adverse effects. For example, in patients with T2D and metabolic dysfunction-associated steatotic liver disease (MASLD), a term that replaces nonalcoholic fatty liver disease (NAFLD), moderate alcohol consumption has been associated with an increased risk of liver fibrosis, likely due to a synergistic effect between alcohol intake and insulin resistance [38]. T2D and MASLD are common aspects of metabolic syndrome, associated with obesity and insulin resistance. MASLD occurs in up to 70% of patients with T2D. A synergistic, bidirectional relationship between T2D and MASLD has been described, meaning that each affects the disease course of the other [39]. For this reason, the issue of MASLD in patients with T2D appears very delicate and must always be carefully evaluated when consuming any type of alcoholic beverage.

Conversely, the UK Prospective Diabetes Study reported an association between alcohol intake and increased risk of diabetic retinopathy in men, though not in women [40]. In contrast, other cohort data [41] suggested a reduced incidence of diabetic retinopathy among moderate drinkers by two-thirds, without worsening diabetic retinopathy in those who already had the disease. Another critical condition is diabetic peripheral neuropathy. Earlier evidence indicates that heavy alcohol consumption exacerbates nerve damage, compounding diabetes-related neuropathy [42].

**Figure 2:**
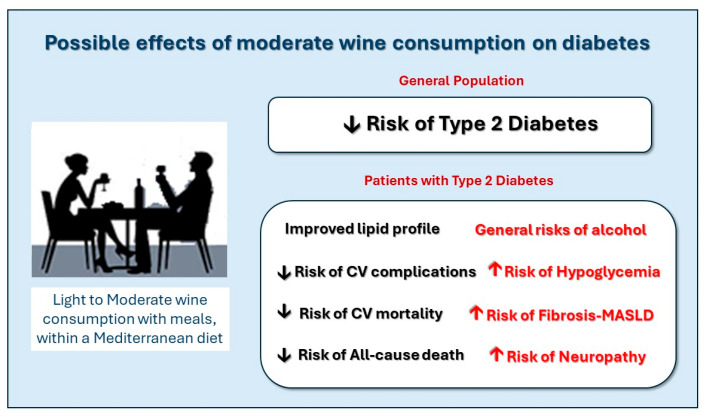
Possible effects of moderate wine consumption on diabetes. Abbreviations: CV: cardiovascular; MASLD: metabolic dysfunction-associated steatotic liver disease.

Overall, the evidence suggests moderate alcohol consumption, particularly wine, may confer CV and metabolic benefits in patients with T2D, especially when consumed within a balanced dietary pattern such as the Mediterranean diet (Figure 2). However, these effects are not uniform for all populations. Careful consideration of individual patient characteristics, including age, sex, comorbidities, liver status, and pharmacological treatment is essential. Further large-scale, well-designed intervention studies are needed to clarify these relationships and to define safe consumption patterns.

**Table 2:** Effects of moderate alcohol/wine consumption in patients with T2D.

Study Design	Results	Effects of Wine	Reference
**Meta-analyses**			
5447 patients in the SMART study (Meta-analysis)	Alcohol consumption was inversely associated with CHD and stroke. Compared with abstainers. Individuals consuming 10–20 drinks/week had an HR of 0.39 for CHD and 0.67 for stroke. Significant U-shaped associations were observed for all-cause death.	Moderate alcohol consumption associated with lower vascular and all-cause mortality. No specific data on wine.	Beulens et al., 2010 [25]
3153 T2D patients (Meta-analysis of 14 RCT)	Pooled data of nine short-term studies showed no difference in blood glucose concentrations. Similarly, pooled data from five medium-term studies found no difference in blood glucose or HbA1c concentrations after 4–104 weeks of moderate alcohol consumption.	No significant impact of light-to-moderate alcohol consumption on glucose metabolism. No specific data on wine.	Hirst et al., 2017 [37]
Patients with T2D (9 randomized intervention studies)	Significant reduction in diastolic blood pressure and total cholesterol in patients with T2D drinking a moderate amount of wine whereas no noticeable differences in glucose parameters, systolic blood pressure, LDL-C, TG and HDL-C were identified.	Moderate wine intake associated with lower diastolic blood pressure and total cholesterol.	Ye et al., 2019 [34]
2068 patients with diabetes (Type 1 and Type 2), including those with cardiovascular complications	Diabetic patients who consumed red wine had significantly lower systolic blood pressure (BP) (mean difference [MD], −1.33; 95% CI: −1.81, −0.85) and diastolic BP (MD −1.31; 95% CI: −1.80, −0.83) than non-drinkers and increased HDL-C.	Moderate red wine consumption associated with lower blood pressure and increased HDL cholesterol.	Naame et al., 2019 [32]
**Randomized Controlled Trials (RCT)**			
115 patients with T2D and with non-fatal myocardial infarction (RCT)	Concentrations of nitrotyrosine, CRP, TNF-alpha, IL-6, and IL-18 were higher in the control group (water) than in the intervention group (red wine). Myocardial performance and ejection fraction were improved in the wine group.	Moderate red wine consumption associated with reduced oxidative stress and pro-inflammatory cytokines.	Marfella et al., 2006 [31]
224 alcohol abstaining adults with well-controlled T2D (2 year RCT)	Red wine (150 mL at dinner) significantly increased HDL-C by 0.05 mmol/L and apolipoprotein A1 by 0.03 g/L, while decreasing the total cholesterol-HDL-C ratio by 0.27.	Red wine associated with positive effects on cholesterol levels.	Gepner et al., 2015 [29]
24 T2D patients (Randomized crossover study)	Red wine increased awake systolic and diastolic BP (*p* = 0.033, *p* = 0.008, respectively) and decreased asleep diastolic BP (*p* = 0.016) compared to water. Red wine increased heart rate but did not affect glycemic control or other CVD risk factors.	Moderate red wine consumption increases awake BP and heart rate and decreases asleep diastolic BP. It does not significantly modify glycemic control and other CV risk factors.	Mori et al., 2016 [33]
48 well-controlled T2D patients (RCT)	Weight loss was similar between groups (red wine 1.3 kg; water 1.0 kg). Changes in abdominal adipose tissue distribution were also comparable.	Wine consumption with dinner did not result in weight gain or increased abdominal adiposity.	Golan et al., 2017 [30]
**Cohort Studies**			
541 white diabetic men (Cohort study)	The prevalence of symptomatic peripheral neuropathy was substantially higher among diabetic men who consumed alcohol excessively.	Excessive alcohol consumption exacerbates nerve damage.	McCulloch et al., 1980 [42]
2964 T2D (Multicenter RCT/Cohort)	In men, higher alcohol intake was associated with more severe retinopathy (*p* < 0.05).	High alcohol intake linked to severe retinopathy in men.	Kohner et al., 1998 [40]
5103 women with T2D (Prospective cohort study)	Compared with diabetic women reporting no alcohol intake, the multivariate adjusted RR for CHD was 0.72 for 0.1–4.9 g/day and 0.45 for >5 g/day of alcohol.	Inverse association between alcohol intake and CHD risk in diabetic women.	Solomon et al., 2000 [21]
287 T2D patients (Prospective cohort study)	Compared with non-drinkers, individuals consuming 16 to 30 g/day exhibited significant reduction in both CHD mortality and all-cause mortality.	Moderate alcohol consumption associated with significant reduction in mortality.	Diem et al., 2003 [22]
3314 patients with T2D (Prospective cohort study)	Moderate consumers had a lower risk of CVD events (aHR: 0.83), microvascular complications (aHR: 0.85) and all-cause mortality (aHR: 0.87).	Associations were more pronounced among T2D participants who predominantly consumed wine.	Blomster et al., 2014 [28]
656 T2D (Population-based cohort of Singaporean Indians)	Reduction in incident diabetic retinopathy (DR) among patients with T2D who consumed alcohol (OR: 0.36) compared with those who did not. No association with DR progression.	Moderate alcohol associated with two-thirds reduced incidence of retinopathy.	Gupta et al., 2021 [41]
3521 patients with T2D or prediabetes (Prospective cohort study)	Occasional alcohol consumption was associated with a reduced 10-year risk of all-cause mortality (RR: 0.51). Heavy alcohol consumption was associated with an increased risk of stroke (RR: 2.503).	Occasional alcohol consumption associated with reduction in all- cause mortality, but heavy intake increases stroke risk.	Cui et al., 2023 [23]
642,359 patients with T2D (Nationwide Korean prospective cohort study)	Compared to non-drinkers, moderate alcohol consumption was associated with reduced all-cause mortality (aHR: 0.81) and cancer mortality (aHR: 0.88). In contrast, heavy drinking was associated with increased mortality.	J-shaped relationship associated with alcohol consumption and mortality.	Lee et al., 2025 [26]
222,334 T2D with 1998 AUD (Retrospective cohort study)	Individuals with AUD (alcohol use disorder) had higher risk of hypoglycemia (aRR: 2.14), cardiovascular complications (aRR: 1.43), and neuropathy (aRR: 1.26).	Excessive alcohol consumption linked to increased complications.	Iturralde et al., 2025 [36]
**Case–Control Studies**			
412 T2D patients (Case–control study)	A J-shape association was observed between alcohol intake and the risk of acute coronary syndrome (ACS). Low alcohol consumption (<12 g/day) was associated with a 47% reduction in ACS prevalence	Low alcohol intake associated with reduction in ACS risk, while high intake increases it.	Pitsavos et al., 2005 [24]
3153 patients with T2D (Case–control study)	Alcohol abuse in adults with insulin-treated T2D was associated with an increased risk for severe hypoglycemia (OR: 2.43)	Alcohol abuse is a significant risk factor for severe hypoglycemia.	Settles et al., 2022 [35]
**Cross-Sectional Studies**			
86 patients with NAFLD, 42 with T2D (Cross-sectional study)	Among patients with NAFLD (Non Alcoholic Fatty Liver Disease) and T2D, moderate alcohol consumption was associated with higher prevalence of advanced fibrosis compared with low consumption	Alcohol may have a synergistic effect with T2D on liver fibrosis in NAFLD patients.	Blomdahl et al., 2021 [38]
26,473 US adults (Cross-sectional study)	Heavy alcohol consumption was associated with a higher risk of diabetic kidney disease (DKD). In contrast, moderate drinking was associated with a lower risk of DKD	Moderate alcohol may be associated with favorable effects on kidney function.	Yang et al., 2025 [27]

## 4. Discussion

Alcohol abuse is well established as a major contributor of severe acute and chronic health conditions [43]. Excessive consumption of alcoholic beverages is strongly associated with an increased risk of CVD, cancer, and digestive disorders, regardless of alcoholic beverage type or drinking pattern [43,44,45]. Additional adverse outcomes include an increased risk of infections, fetal harm, vehicle accidents, and violent behavior [46,47,48].

With regard to the risk of developing T2D, substantial evidence indicates that heavy alcohol consumption is associated with an increased risk [49,50,51]. However, whether moderate wine consumption can modulate the risk of developing T2D and whether patients with diabetes can safely consume wine in moderation remains a matter of ongoing debate. A key consideration is the relatively low alcohol content of wine and its high concentration of bioactive compounds, particularly polyphenols, such as flavonoids, which are more abundant in wine, especially red wine, than beer or spirits.

Regarding the first question, the analysis of the data compiled in this review highlights that moderate alcohol consumption does not increase, and may modestly reduce, the risk of developing T2D. Notably, moderate wine consumption appears to be associated with a greater reduction in T2D risk, particularly when consumed with meals. Several studies reported a dose-dependent relationship between alcohol intake and T2D risk, typically characterized by a J-shaped curve, with the lowest risk observed at moderate levels of consumption [8]. In several studies, risk reduction has been estimated at approximately 18–20%, although this varies depending on the type of alcoholic beverage considered. Moderate intake of wine or beer (20–30 g/day of alcohol) has been associated in some studies with risk reduction of around 20%, whereas spirits (7–15 g/day) confer a smaller favorable association (~5%) [49]. Some studies reported that the frequency of consumption matters, showing that regular low consumption is more favorably associated than the same amount over few days [17].

The potentially greater association with favorable effects of wine as compared to other alcoholic beverages is likely attributable to its high polyphenol content. Resveratrol has demonstrated beneficial metabolomic effects in experimental models, including improved glycemic control via activation of sirtuins [52]. In addition, polyphenols exert antioxidant and anti-inflammatory effects, mitigating oxidative stress associated with dietary and environmental toxins [53,54].

Based on approximate ranges observed in several studies the moderate wine consumption could be defined as 10–15 g/day of alcohol for women (equivalent to one glass of 125–150 mL of wine) and 15–30 g/day for men (up to two glasses). In those studies, these levels are associated with potentially favorable metabolic and cardiovascular effects, while minimizing harm. However, it must always be considered that the J-shaped relationship between alcohol/wine consumption and T2D may be influenced by residual confounding, healthy user bias, former drinker bias (sick-quitter effect), self-reporting bias, lifestyle patterns, socioeconomic factors, dietary habits, physical activity, and reverse causation. In any case these values should not be interpreted as a recommended target, but only as an upper exposure range reported in some studies. From a clinical perspective, particularly in patients with T2D, the safest interpretation is that alcohol intake should remain as low as possible.

Another aspect to consider is that the potential association of wine with favorable effects appears to be enhanced when consumption occurs with meals [7], likely due to slower alcohol absorption and reduction in postprandial oxidative stress and inflammatory state [32,55,56]. The anti-inflammatory effect of wine polyphenols has been widely described in many scientific observations [57,58]. Further studies on this topic are necessary to acquire conclusive information.

Lifestyle factors may contribute to these observed associations. Individuals who consume wine moderately often exhibit healthier dietary patterns and lifestyle behaviors compared with abstainers or heavy drinkers [59]. Overall, based on many observational epidemiological studies, it can be hypothesized that individuals who consume wine in moderation, especially with meals, can have a reduced risk of developing T2D. It must be emphasized that this association today must be distinguished from the concept of causation due to the absence of randomized evidence.

Regarding the second question of this review, in individuals with established and well-controlled T2D, in the absence of significant comorbidities and vulnerability, moderate alcohol consumption, particularly wine, may be associated with some positive effects. Evidence suggests reductions in CV events (heart attacks and stroke), T2D-related nephropathy, improved lipid profiles, anti-inflammatory effects, and potential association with favorable gut microbiota composition [60]. Polyphenols in red wine may inhibit LDL oxidation and platelet aggregation, thereby contributing to antiatherogenic and antithrombotic effects [61]. Short-term studies indicate that moderate alcohol consumption can favorably alter blood lipids by increasing HDL cholesterol and apolipoprotein A-1 [62]. Alcohol has antithrombotic properties by decreasing platelet aggregation and fibrinogen [62]. In addition, some pathways show that light-to-moderate alcohol intake can improve glucose control and reduce markers of systemic inflammation (e.g., IL-6) [62]. However, the extent to which these potentially favorable associations are attributable to alcohol itself or to other components of wine, mainly polyphenols, has yet to be clearly established. The effect of moderate red wine consumption on the gut microbiota has been related to an increase in specific bacterial strains, particularly *Bifidobacteria* and *Prevotella* [63]. These changes may contribute to reduced lipopolysaccharides levels, thereby lowering the risk of insulin resistance and exerting favorable effects on T2D and metabolic syndrome [63].

Moderate wine consumption has also been associated with improvements in glycemic controls. Studies indicate reductions in fasting glucose, insulin levels, and insulin resistance (as measured by the HOMA index), without significant effects on postprandial glucose levels [64]. Additional findings include reductions in markers of renal damage, indicating a potential role in slowing progression of diabetic nephropathy [65].

The potential favorable association of moderate wine consumption with some aspects of the health of patients with T2D appears to be particularly pronounced within the context of a Mediterranean dietary pattern. This diet pattern emphasizes olive oil as the main source of fat, plant-based foods, nuts, fish, poultry, dairy products, low consumption of red meat and moderate consumption of wine in meals over the week, avoiding binge drinking [66]. The Mediterranean-style eating pattern is recommended by major organizations, including the American Diabetes Association (ADA), the American Heart Association (AHA), the European Association for the Study of Diabetes (EASD), for improving glycemic control and reducing CVD risk [67,68,69].

Adherence to this dietary pattern may produce synergistic antioxidant and anti-inflammatory effects from wine polyphenols and various components of the Mediterranean diet, which may enhance insulin sensitivity in peripheral tissues and improve vascular endothelial function, thereby reducing the risk of heart failure and CVD complications [70].

The evaluation of various studies reported in this review suggests that moderate wine consumption during meals may be associated with an improvement in glycemic control without significantly affecting body weight or causing major adverse effects. Several studies indicate a greater advantage with red wine compared to white wine in this aspect, likely due to its higher polyphenol content. However, the lack of randomized trials and of intervention studies on large number of people makes this observation a hypothesis and not definitive evidence. In individuals with T2D or dysglycemia/prediabetes, it is also important to avoid sweet and straw wines, given their high sugar content [71].

Despite these potential positive associations, important risks should be considered. Alcohol consumption, particularly at high levels, increases the risk of hypoglycemia, especially in elder individuals receiving insulin or sulfonylureas [72]. That risk may persist several hours after alcohol consumption, especially overnight. Nocturnal hypoglycemia is of particular concern, as alcohol can impair hepatic glucose production and alter hormonal responses. Although a large meta-analysis has not found an increased risk of hypoglycemia with moderate alcohol intake (~20 g/day), caution is warranted, and patients should be advised to consume alcohol only with meals and within recommended limits [36]. Patients should always be informed of the need to limit consumption of alcoholic beverages, if they are taking antidiabetic therapy and should absolutely avoid taking alcohol and antidiabetic drugs without following a regular meal [73].

Furthermore, alcohol consumption may be contraindicated in certain subgroups of patients with T2D, particularly those with metabolic dysfunction-associated steatotic liver disease (MASLD), advanced neuropathy, or other major complications. MASLD deserves special attention because it is highly prevalent in people with T2D. MASLD, advanced fibrosis, significant liver disease, and history of alcohol-related vulnerability should be considered contraindication and strong reasons for abstinence. The association between alcohol use and diabetic retinopathy remains uncertain and warrants further study. Therefore, before consuming any alcoholic beverage, patients with T2D should undergo an individualized medical assessment to confirm that their diabetes is well-controlled and that no comorbidities clearly contraindicate alcohol use.

In addition, alcohol consumers should be advised about the risks of excessive alcohol consumption, such as alcohol dependence, several types of cancer, cardiomyopathy, and advanced hepatic and neuro-psychiatric diseases. The potential advantages of alcohol or wine on this population are restricted to low consumptions and should be counterbalanced with the individual risks of creating dependence and alcohol-related complications.

Finally, in this review, several limitations should be acknowledged. Variability in genetic background, socioeconomic status, dietary habits and lifestyle, as well as study design and sample size may influence the observed associations [74,75]. Additionally, many studies do not adequately distinguish between types of alcoholic beverages, making difficult to isolate the specific effects of wine, particularly red wine, from those of beer and spirits [74]. Other limitations are the impossibility of generalizing the results to the Asian population due to differences in genetic metabolism (e.g., ALDH2 deficiency) and dietary patterns; the narrative review design of this paper lacking a formal risk of bias assessment; the variability in the definitions of “moderate” consumption; the general bias of self-reported alcohol intake; and the fact that most of the data is based on observational studies.

## 5. Conclusions

T2D is a highly prevalent disease worldwide and is associated with a substantially increased risk of complications, particularly CVD events, leading to reduced quality of life, increased disability, and higher mortality.

The risk of developing T2D can be reduced through healthy lifestyle habits, including balanced diet, regular physical activity, and appropriate weight management that constitute well established preventive strategies. In addition to these measures, in some studies, moderate alcohol consumption, particularly wine, has been associated with a lower risk of developing T2D. This risk reduction seems more pronounced when wine, especially red wine, is consumed with meals. Some of these possible associations are likely related to polyphenols, which may exert favorable effects on carbohydrate metabolism. Evidence from multiple studies across diverse populations suggests that moderate wine consumption may confer advantages compared with both abstention and excessive drinking, even though these results are mostly based on observational studies. This implies associative and not causal evidence and therefore cannot be promoted as a preventive recommendation.

Moderate consumption of alcoholic beverages, particularly wine, may also be associated with clinically favorable effects in individuals with established T2D. These include a reduced risk of secondary complications of T2D, such as CVD, stroke, nephropathy, and renal failure. These effects are partially due to the non-alcoholic components of wine, especially polyphenols, which exhibit antioxidant, anti-inflammatory, antiatherogenic, and favorable metabolic properties. Such possible advantages may be further enhanced when wine is consumed as part of a Mediterranean-style diet and drinking pattern, owing to synergistic biological effects. In these conditions the possible favorable associations of wine consumption cannot be completely separated from the broader dietary and lifestyle context of some Mediterranean dietary patterns.

However, in patients with T2D, alcohol consumption is not without risks. Alcohol may induce hypoglycemia, particularly in type 1 diabetes and in diabetics receiving insulin or sulfonylureas, and especially when consumed in excess or without adequate food intake. Moreover, consumption of alcoholic beverages should be avoided in specific subgroups of patients with T2D, including those with MASLD or other liver diseases, peripheral neuropathy, psychiatric disorders, acute and vulnerable conditions, or during breastfeeding.

Given the well-established adverse effects of excessive alcohol consumption, patients with T2D who do not drink alcohol should not start. For those who already consume alcoholic beverages, in the absence of liver disease, peripheral neuropathy, psychiatric disorders and vulnerable conditions, caution should always be considered, and alcohol intake should remain moderate, that is as low as possible, after individualized clinical assessment. The alcohol should preferably be wine, always consumed with meals and within a Mediterranean style dietary pattern (Table 3).

## Figures and Tables

**Table 3:** Guidelines for wine consumption in patients with T2D.

1. If you have T2D do not start drinking if abstinent.
2. If already drinking, preferably take wine in moderation (as low as possible), when T2D is well-controlled, and always with meals, following a healthy dietary pattern like Mediterranean-style, and after individualized clinical assessment. Avoid alcohol in vulnerable patients.
3. Regularly monitor your blood sugar. Alcohol can cause a drop in blood sugar several hours after drinking, especially if you take antidiabetic medications. Avoid drinking in excess or on an empty stomach.
4. Individual responses vary. Some patients with T2D must avoid alcohol consumption due to specific therapies or other concomitant diseases like neuropathy, liver disease, psychiatric disorders or addictions and alcohol-related vulnerability. For this reason, always talk to your doctors before drinking alcohol/wine and follow their therapeutic and behavioral recommendations.

## Data Availability

No new data were created or analyzed in this study. Data sharing is not applicable to this article.

## Abbreviations

The following abbreviations are used in this manuscript:

ACS	Acute Coronary Syndrome
ADH1B	Alcohol Dehydrogenase 1B
aHR	Adjusted Hazard Ratio
BMI	Body Mass Index
BP	Blood Pressure
CHD	Coronary Heart Disease
CI	Confidence Interval
CRP	C-Reactive Protein
DBP	Diastolic Blood Pressure
DKD	Diabetic Kidney Disease
DRW	Dealcoholized Red Wine
EPIC	European Prospective Investigation into Cancer and Nutrition
HDL-C	High-Density Lipoprotein Cholesterol
HR	Hazard Ratio
IL	Interleukin (e.g., IL-6, IL-18)
LDL	Low-Density Lipoprotein
MeSH	Medical Subject Headings
NHANES	National Health and Nutrition Examination Survey
OR	Odds Ratio
PRISMA	Preferred Reporting Items for Systematic Reviews and Meta-Analyses
RCT	Randomized Controlled Trial
RR	Relative Risk
SANRA	Scale for the Assessment of Narrative Review Articles
SBP	Systolic Blood Pressure
T2D	Type 2 Diabetes
TNF-α	Tumor Necrosis Factor-alpha
WHO	World Health Organization
